# Research on Piezoelectric Guided Wave Frequency Diverse Array-Based Damage Location Method for Thin-Walled Structures

**DOI:** 10.3390/mi16101172

**Published:** 2025-10-16

**Authors:** Changlin Wang, Quanyao Hu, Yongteng Zhong

**Affiliations:** 1 School of Information Technology, Jiangsu Open University, Nanjing 210036, China; wangcl@jsou.edu.cn; 2 College of Mechanical and Electrical Engineering, Wenzhou University, Wenzhou 325035, China; 20451438003@stu.wzu.edu.cn

**Keywords:** guided waves, frequency diverse array, damage location, MUSIC

## Abstract

Phased array technology can be realized with directional control with fixed beam steering. However, its directionally dependent beam pattern limits the efficiency of suppressing undesirable distance interference. This paper presents a guided wave frequency diverse array-based damage location method for thin-walled structures. Firstly, a guided wave frequency diverse array signal model is derived with a relatively small frequency increment that can achieve distance–direction two-dimensional focusing. Secondly, three types of receiving arrays, including a monostatic array, following array, and symmetric array, are constructed to achieve the maximum damage-induced signal amplitude. Finally, a two-dimensional multiple signal classification (MUSIC)-based damage location method is applied for damage imaging in thin-walled structures. Simulations on an aluminum plate and the experiments on an epoxy laminate plate demonstrate the validity and effectiveness of the proposed method.

## 1. Introduction

The accurate quantification of damage by using structural health monitoring (SHM) methods in service is very important to prognose thin-walled structures [1]. Ultrasonic guided waves, such as Lamb waves, can propagate over large distances in thin-walled plates [2]. Lamb waves have been increasingly employed to develop various SHM techniques, and they have shown usefulness in locating and quantifying damage in plate-like and shell-like structures.

When active Lamb waves are incident on damage, scattering will happen in all directions [3]. To collect these scattered waves in real time, an array composed of a certain number of sensors is integrated into the structure. As a common arrangement form, sparse arrays have a relatively large distance between two adjacent sensors during the arrangement process, usually between ten and twenty centimeters. There are many common damage location methods based on sparse arrays, such as time-reversal focusing, delay and sum, and tomography imaging, etc. Yu et al. [4] proposed a rapid two-dimensional finite-difference time-domain method for damage detection in stiffened plates using time-reversed Lamb waves. Nokhbatolfoghahai et al. [5] evaluated the performance of the sparse reconstruction and the delay-and-sum methods for damage localization under various environmental and operational conditions. Ling et al. [6] developed a wide-band dispersion reversal method-optimized tomography. Overall, the above methods based on sparse array arrangements may be able to precisely identify damage information in a certain local area by reducing the distance between adjacent sensors. However, when it comes to damage monitoring on large or complex structures, a large number of physical sensors need to be distributed throughout the entire area.

In recent years, a new physical sensor arrangement method called dense array has emerged. The distance between two adjacent sensors is very small, usually several to tens of millimeters, which makes it more suitable for large or complex structures. As one of the most representative methods in dense arrays, the ultrasonic guided wave phased array method achieves directional scanning by superimposing the excitation signal beam in a specific direction through phase control. Yuan et al. [7] investigated the influence of different curvature radii in the curved plate on the guided wave propagation behavior of the phased array, and proposed a phased array focusing method for the location of damage on the curved plate. Aimed at utilizing Lamb wave information contained in multiple frequency bands to improve the image quality of Lamb wave phased array imaging, Xu et al. [8] proposed a multi-narrowband fusion method for an aluminum plate with two defects. Gao et al. [9] proposed a phased array imaging method via dual-frequency fusion for compensating the grating lobe effect produced by high-frequency narrowband excitation pulses. Yang et al. [10] pointed out that it is urgently necessary to solve the bottleneck problem of the rapid attenuation of guided wave energy, and to enhance the reliability of traditional ultrasonic guided wave phased array technology in long-distance damage detection of large structures. According to these studies, phased array technology can be achieved only in the direction domain with fixed beam steering direction for all distances, which limits its applicability in suppressing undesirable distance-dependent interference [11].

Recently, a flexible array called frequency diverse array has been introduced as having a small frequency and successfully achieves distance–direction-dependent beam patterns. Lang et al. [12] introduced a frequency diverse array from radar to Lamb waves to realize the angle-range focusing on damage localization, and its superiority was validated by an experiment on an aluminum plate with multiple adjoining surface and inner defects. Obviously, the frequency diverse array activated as the transmitter, making it a good choice for damage detection. The design of the receiver is also important for damage imaging. A statistical super-resolution technique called the multiple signal classification (MUSIC) algorithm published in *Nature Communications* had a comparable or better performance in comparison with other localization techniques [13]. Yuan et al. [14] proposed a dual-array-based guided wave MUSIC focusing method with anisotropy compensation abilities to address the problem of damage imaging for complex composites. The authors conducted a series of studies on this algorithm and developed two-dimension MUSIC-based multi-damage location method [15].

This paper presents a guided wave frequency diverse piezoelectric sensor array-based damage location method for thin-walled structures. The layout of the paper is organized as follows: Section 2 introduces a frequency diverse piezoelectric sensor array-based damage location method. In Section 3 and Section 4, numerical and experimental verification on an aluminum plate and epoxy laminate plate are performed, and Section 4 gives the conclusion.

## 2. Frequency Diverse Piezoelectric Sensor Array-Based Damage Location Method

### 2.1. Frequency Diverse Array Signal Transmitter Model

**Figure 1:**
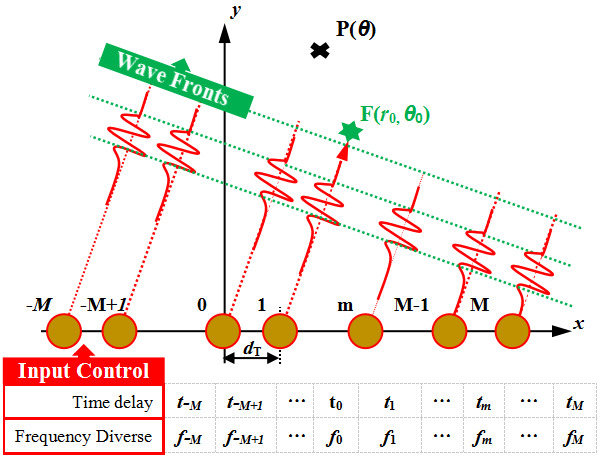
Schematic of a frequency diverse array.

A uniform linear array was constructed using piezoelectric sensors, marked as −*M*, −*M*+1, …, to *M*, with a distance of *d*_T_ between the array elements. The array center element marked as 0 is used as the origin to establish the coordinate system, as seen in Figure 1. In SHM applications, the five-wave peak narrowband signal transmitted by the *n*-th sensor is expressed as

(1) $$s(t)=\exp(-j2\pi f_{0}t)$$

where *f*_0_ is the center frequency of the signal. Each piezoelectric sensors is an omnidirectional wave source, the excitation signal *s*(*t*) is assumed to be transmitted from the origin to any search point P(*θ*), and the signal can be expressed in the form of a complex envelope as

(2) $$s_{p}(t)=\frac{1}{\sqrt{r}}s(t-\frac{r}{c})$$

When the P(*θ*) is far from the sensor array, the far-field parallel-ray approximation has been proven to be applied, and the signal received at the search point P(*θ*) is

(3) $$s_{m}^{P}(t)=\frac{1}{\sqrt{r}}\sum_{m=-M}^{M}s(t-\frac{r_{m}}{c})=\frac{1}{\sqrt{r}}\sum_{m=-M}^{M}s(t-\frac{r-md_{T}\cos\theta }{c}),m=-M,\ldots ,M$$

where r_m_ is the target slant range for the *m*-th sensor and c is the speed of the Lamb wave. The phased arrays can achieve focusing and beamforming using the time delays $\Delta_{m}$ of the *m*-th sensor, and Equation (2) becomes

(4) $$s_{p}(t)=\frac{1}{\sqrt{r}}\sum_{m=-M}^{M}s(t-\frac{r}{c}+\frac{md_{T}\cos\theta }{c}-\Delta_{m})$$

**Figure 2:**
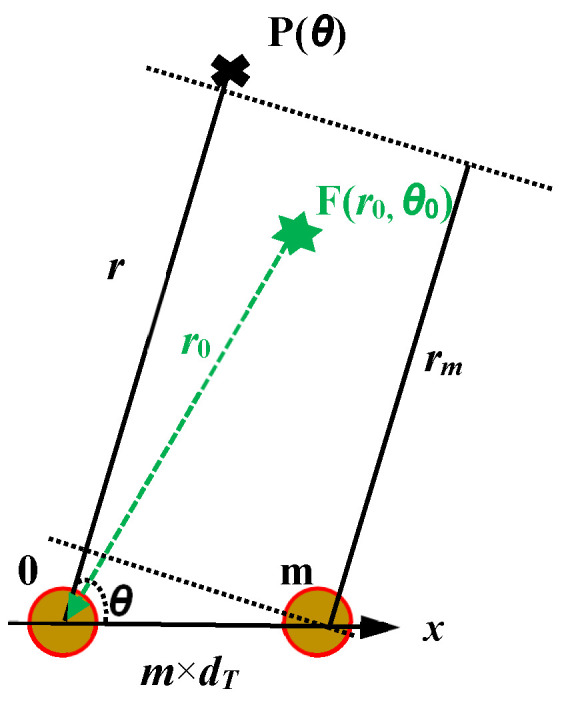
Direction-dependent and distance–direction-dependent beam pattern.

Hence, as seen in Figure 2, when time delays of the array element are equal to the differences in arrival time, all the array sensor signals will arrive at P(*θ*) at the same time. That is,

(5) $$\Delta_{n}=\frac{md_{T}\cos\theta }{c}$$

When searching the unknown target direction θ, the whole space should be swept by varying the direction θ through changes in the time delay. Obviously, conventional phased arrays provide only direction-dependent beam patterns.

In a frequency diverse array, there is a small frequency increment Δf between adjacent array elements and this results in a distance–direction-dependent beam pattern F(*r*, *θ*). Hence, the center element with frequency $f_{0}$ is taken as the reference; then, the carrier frequency of the *m*-th sensor can be presented as

(6) $$f_{n}=f_{0}+m\Delta f$$

Here, the five-wave peak narrowband signal transmitted by the *n*-th sensor is rewritten as

(7) $$s_{m}(t)=\exp(-j2\pi f_{m}t)$$

and the signal received at the search point F(r, θ) is

(8) $$\begin{array}{l} s_{m}^{F}(t)=\frac{1}{\sqrt{r_{0}}}\sum_{m=-M}^{M}\exp\left\{-j2\pi f_{m}(t-\frac{r_{m}}{c})\right\}\\ =\frac{1}{\sqrt{r_{0}}}\sum_{m=-M}^{M}\exp\left\{-j2\pi (f_{0}+\Delta f)(t-\frac{r_{0}-md_{T}\cos\theta_{0}}{c})\right\} \end{array}$$

Then, the phase of the 0-th sensor is

(9) $$\phi_{0}=2\pi f_{0}\frac{r_{0}}{c}$$

And the phase of the *n*-th sensor is

(10) $$\phi_{m}=\frac{2\pi }{c}\left[f_{0}+m\Delta f\right]\left[r_{0}-md_{T}\cos\theta_{0}\right]$$

The phase difference between the *n*-th element of the excitation of the frequency diverse array and the reference element reaching the target can be expressed as

(11) $$\begin{array}{ll} \Delta \phi_{m0} & =\phi_{m}-\phi_{0}\\ & =\frac{2\pi }{c}\left[f_{0}+m\Delta f\right]\left[r_{0}-md_{T}\cos\theta_{0}\right]-\frac{2\pi }{c}f_{0}r_{0}\\ & =\frac{2\pi }{c}\left[-f_{0}md_{T}\cos\theta_{0}+mr_{0}\Delta f-m^{2}\Delta fd_{T}\cos\theta_{0}\right] \end{array}$$

If $\Delta f<<f_{0}$, $2\pi m^{2}\Delta fd_{T}\cos\theta /c<<1$. Therefore, the quadratic term in the previous expression can be ignored.

(12) $$\Delta \phi_{m0}\approx m\times \frac{2\pi }{c}\left[-f_{0}d_{T}\cos\theta_{0}+r_{0}\Delta f\right]$$

Let

(13) $$\Theta (r_{0},\theta_{0})=\frac{2\pi \left[f_{0}d_{T}\cos\theta_{0}-r_{0}\Delta f\right]}{c}$$

And define the transmit steering vector $a(r_{0},\theta_{0})$ as

(14) $$a^{F}(r_{0},\theta_{0})={\left[\begin{array}{ccccccc} e^{-jM\Theta \left(r_{0},\theta_{0}\right)} & \ldots & 1 & \ldots & e^{-jn\Theta \left(r_{0},\theta_{0}\right)} & \ldots & e^{jM\Theta \left(r_{0},\theta_{0}\right)} \end{array}\right]}^{T}$$

where T denotes the transpose operator. When the frequency increment is a fixed value, the transmit steering vector is a function of the target direction and distance. Compared with the one-dimensional direction search of phased arrays, frequency diverse arrays have distance–direction-dependent beam patterns.

### 2.2. Two-Dimensional MUSIC-Based Damage Location

**Figure 3:**
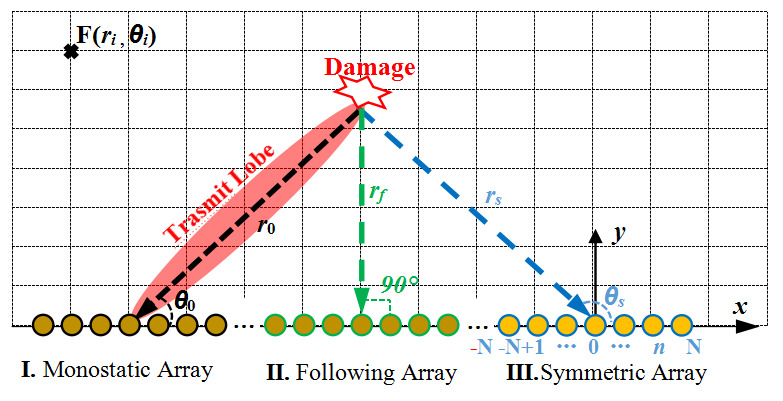
Three types of receiving array constructions.

When a frequency diverse array is activated as the transmitter, as seen in Figure 3, the Lamb waves reflected by damage located at F(*r_i_*, *θ_i_*) are received at all the sensors of one array. When the damage distribution is perpendicular to the main lobe, the damage echo will be the maximum energy. However, due to the different orientations of the damage, the energy distribution of the reflected waves in various directions are quite different. Theoretically, a continuous and uniform linear array along a certain direction of the monitored structure can be arranged, such as a large-scale sensing–monitoring integrated smart skin with ultra-low weight and ultra-low power consumption [16]. Here, it is defined as a chain array. Then, a subarray containing (2*M*+1) array elements can be defined as a receiving array.

Here, three types of receiving arrays are constructed, including a monostatic array, following array, and symmetric array. Among them, the following array is an array that changes with the alteration of the scanning position, and the symmetric matrix is a symmetric array with the scanning position as the axis. Three arrays are arranged in a straight line, which is convenient for processing and installation. Moreover, damage detection at different positions can be switched among each other to achieve the maximum damage echo signal.

Theoretically, a uniform linear array can be arranged in a certain direction in the monitored structure. Then, a subarray of (2*M*+1) consecutive array elements can be defined as a receiving array, and the array parameters are consistent with those in Section 2.1. The corresponding received steering vector $b(r_{D},\theta_{D})$ can be written as

(15) $$b(r_{S},\theta_{S})={\left[\begin{array}{ccccccc} e^{-j2\pi f_{0}\frac{r_{-M}-r_{S}}{c}} & \ldots & 1 & \ldots & e^{-j2\pi f_{0}\frac{r_{n}-r_{S}}{c}} & \ldots & e^{-j2\pi f_{0}\frac{r_{M}-r_{S}}{c}} \end{array}\right]}^{T}$$

Let

(16) $$\begin{array}{l} \tau_{m0}=\frac{r_{m}-r_{S}}{c}=\frac{\sqrt{r_{S}^{2}+m^{2}{d_{T}}^{2}-2r_{S}d_{T}\cos\theta_{0}}-r_{S}}{c}\\ \approx \frac{\left(-d_{T}\cos\theta_{0}\right)}{c}m+\frac{\left(-{d_{T}}^{2}\sin^{2}\theta_{0}\right)}{cr_{S}}m^{2} \end{array}$$

In the authors’ previous study [15], two-dimensional MUSIC-based damage location is derived in detail. The estimated location of the damage can be obtained through the following formula as

(17) $$P_{2D-\mathrm{MUSIC}}(r_{S},\theta_{S})=\frac{1}{b^{H}(r_{S},\theta_{S})U_{N}{U_{N}}^{H}b(r_{S},\theta_{S})}$$

where H denotes the complex conjugate transpose, and $U_{N}$ denotes the noise subspace, which is obtained by the eigenvalue decomposition of the covariance vector $\hat{R}$ of the received signal X(t) as

(18) $$\hat{R}=\frac{1}{N}X(t)X^{H}(t)$$

where *N* is the number of the subarray received signal snapshots.

For the symmetric array, the distance and direction of the damage in local coordinates can be obtained as

(19) $$\left(r_{S},\theta_{S})=\arg\;\max P(r_{S},\theta_{S}\right)$$

And the distance and direction of the damage in global coordinates is

(20) $$\left\{\begin{array}{l} r_{D}=r_{0},\theta_{D}=\theta_{0},\mathrm{for}\mathrm{monostaic}\mathrm{array}\\ r_{D}=r_{f}/\sin\theta_{0},\theta_{D}=\theta_{0}+(\theta_{f}-90{}^{\circ}),\mathrm{for}\mathrm{following}\mathrm{array}\\ r_{D}=r_{S},\theta_{D}=\pi -\theta_{S},\mathrm{for}\mathrm{symmetric}\mathrm{array} \end{array}\right.$$

## 3. Simulation Results and Discussion

A finite element analysis model is created using the ABAOUS. The model is a 1000 mm × 1000 mm × 2 mm aluminum plate with four sides fixed. And the mechanical parameters are $\rho =2730{\;\mathrm{kg}/m}^{3}$, E=72 GPa. A C3D8I three-dimensional solid element is selected to discretize this aluminum plate, and the element size is set to 2 mm.

The center of the structure is defined as the coordinate origin. This origin is taken as the reference and 7 nodes are extracted at equal intervals on both sides for loading concentrated dynamic loads as a transmit array, and the spacing $d_{T}$ of adjacent nodes is 10 mm. The excitation frequency $f_{0}$ of the reference array element is selected as 50 kHz, and the frequency increment Δf is set as 1 kHz.

In order to study the two-dimensional focusing detection ability of the array at the damage location F(*r_i_*, *θ_i_*), small holes with the size 2 mm × 1 mm were used to simulate the damage of the structure in this finite element simulation. This simulated damage was arranged at seven representative locations, respectively, which are selected and listed in Table 1.

According to the schematic diagram in Figure 3, three receiving arrays, including a monostatic array, following array, and symmetric array, were calculated and extracted, and a comparative study of array signals and damage imaging was conducted. The time step is 10^−7^ s and the sampling time is 0.7 ms.

### 3.1. Transmitting–Receiving Array Signals

**Figure 4:**
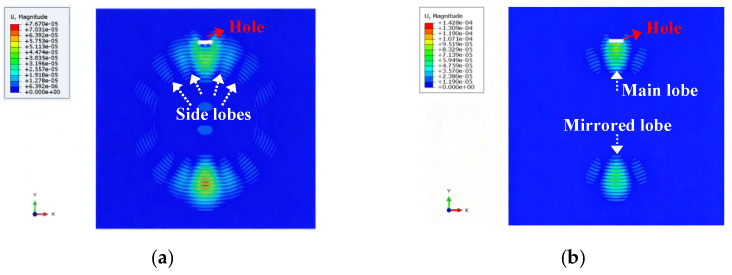
Beamforming characteristic comparison: (**a**) conventional phased array; (**b**) frequency diverse array.

The beamforming characteristics of the signals transmitted between the traditional phased array and the proposed frequency diverse array were first compared and studied. The transmitted array parameters and transmitted signal parameters of the reference elements were consistent, and the simulated damage is a through-hole located at (200 mm, 90°). As seen in Figure 4, both in the phased array and frequency diverse array, a mirrored lobe will occur due to the inherent characteristics of the linear array. The beamforming of the conventional phased array has several side lobes and focuses only the transmitted energy in the direction dimension, but the frequency diverse array hardly has side lobes and focuses better on two-dimensional point (200 mm, 90°).

**Figure 5:**
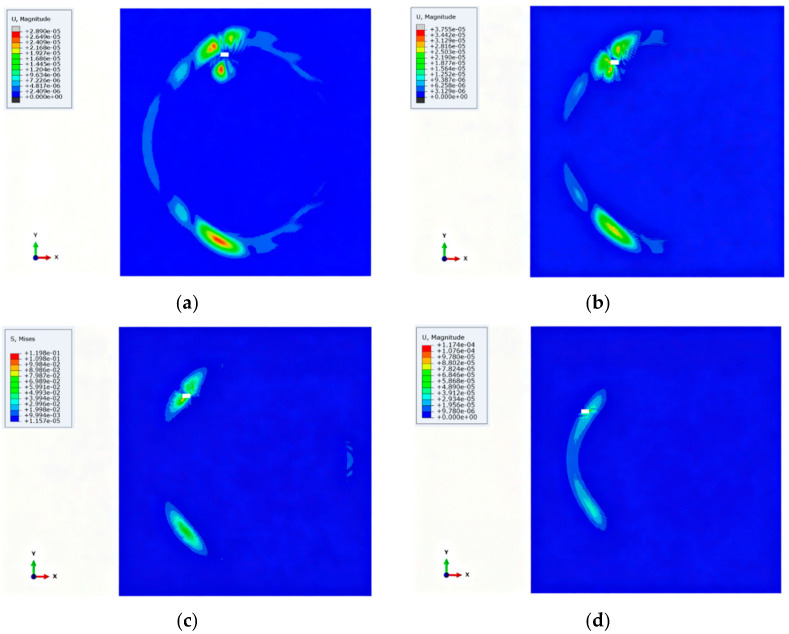
Transmission beamformings in different directions: (**a**) (300 mm, 105°); (**b**) (300 mm, 120°); (**c**) (300 mm, 135°); (**d**) (300 mm, 150°).

The linear array is proven to not have a complete 180° inspection range. When the main lobe becomes closer to 180°, the back lobe will start increasing. To study the detection performance of the frequency diverse array in a different direction, four simulated areas of damage at different positions were selected for comparison. Transmission beamformings in different directions are shown in Figure 5. Most of the energy can be focused on the damage effectively; however, as the damage direction approaches 180°, the focused energy value shows a decreasing trend.

**Figure 6:**
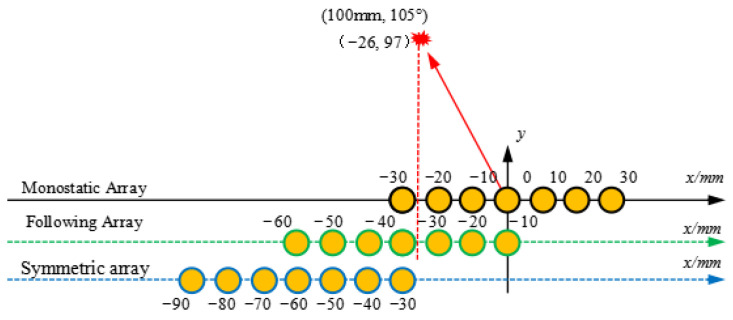
The position coordinates of the three arrays relative to the focus point.

**Figure 7:**
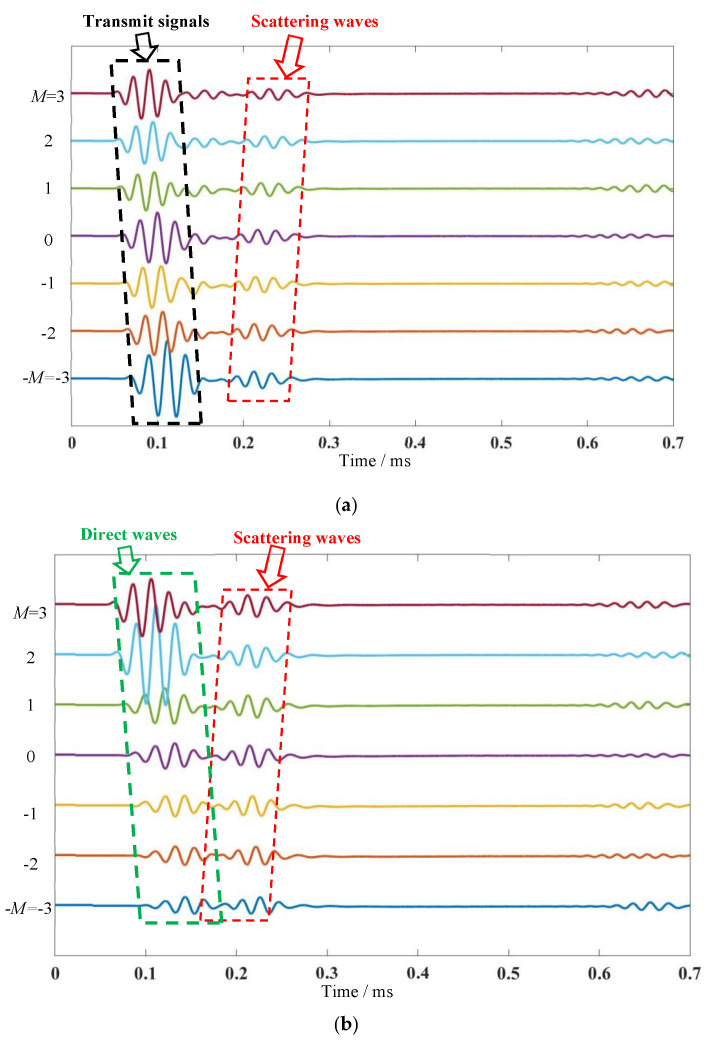

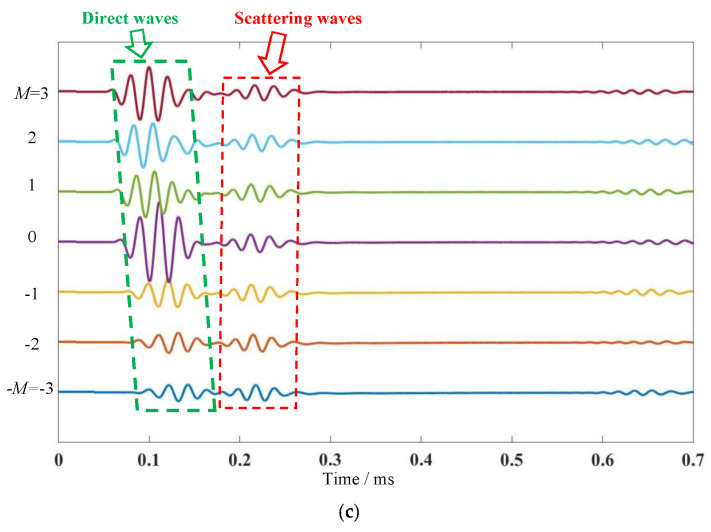
Three types of receiving array signals: (**a**) monostatic array; (**b**) symmetric array; (**c**) following array.

To observe the damage scattering signal more intuitively and taking the damage point (100 mm, 105°) as an example, three arrays are shown in Figure 6. Since the sensors are arranged at equal distances of 10 mm, the coordinates of the center point of the array need to be slightly offset to receive the signals. For the monostatic array, it can transmit signals and receive the scattering wave from the damage, as seen in Figure 7a. A set of array transmit signals modulated with small frequency increments on the basis of the reference 0th element is first presented, and there is a distinct wave front from 0.2 to 0.25 ms. For the multi-input and multi-output mode, an independent receiving array is required. As shown in Figure 7b, the direct waves and damage echo signal received by the array are symmetrical to the transmit array. Figure 7c shows the direct waves and damage echo signal received by the array, which correspond to the focal point coordinates. It is worth noting that the wavefront of the following array basically presents a straight line perpendicular to the *x*-axis.

### 3.2. Damage Imaging Results

**Figure 8:**
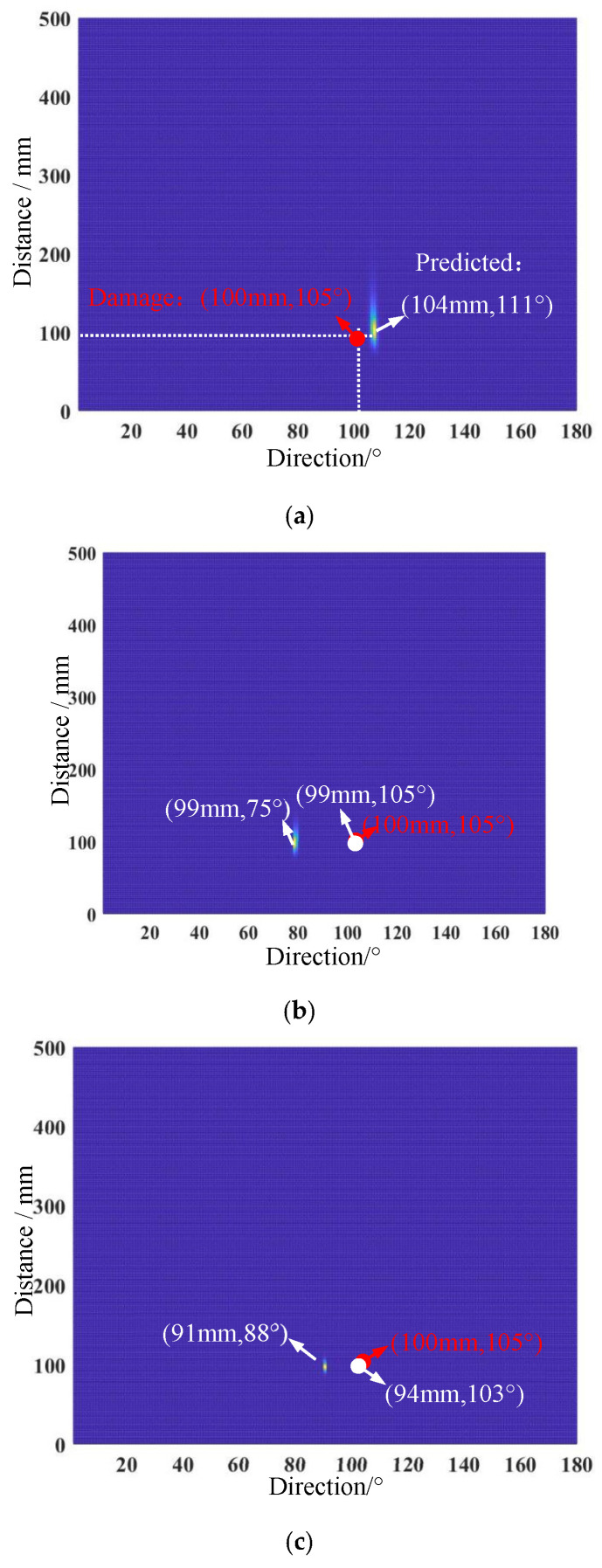
Damage imaging results of three arrays: (**a**) monostatic array; (**b**) symmetric array; (**c**) following array.

The previous section has shown that the scattering waves induced by simulated damage could be detected accurately with the help of a frequency diverse array. For the typical signals shown in Figure 8, damage imaging was performed using the two-dimensional MUSIC-based damage location method presented in Section 2.2. Firstly, the scattering waves received from the monostatic array are intercepted, and then the covariance is calculated to obtain the noise subspace, and the damage imaging can finally be performed using Equation (15). As seen in Figure 8a, a bright pixel point can be easily spotted at the position (104 mm, 111°) which is the position predicted for the simulated damage. Similarly, the damage imaging of the scattering waves received from the symmetric array and following array can be obtained and are shown in Figure 8b,c. Two bright pixel points appeared at the positions (99 mm, 75°) and (91 mm, 88°). According to Equation (18), we obtained the final predicted position coordinates as (99 mm, 105°) and (94 mm, 103°).

**Figure 9:**
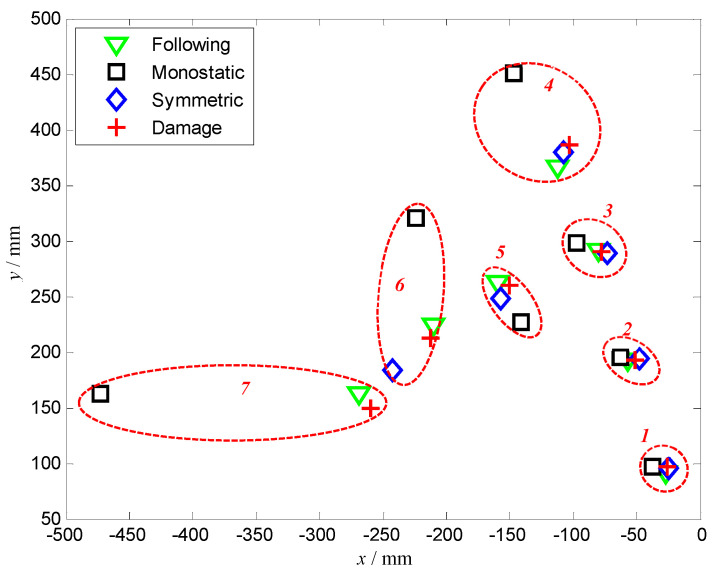
Predicted position for simulated damage.

Damage imaging was performed on all of the simulated damage cases presented in Table 1, and a schematic diagram of the prediction results is shown in Figure 9. For Case 1 to Case 4, the four damage positions are in the same direction but at different distances. It can be found that the predicted damage positions are all consistent with the simulated positions as the distance increases, except that the monostatic array result of Case 4 has a relatively large error, with an approximate 4 cm error in the x direction and 6 cm error in the y direction. For Case 3, Case 5 and Case 6, the four damage positions are at the same distances but in different directions. It is found that the errors of the monostatic array can be predicted.

The results show a sharp rise as the direction increases, and failure at around 150°. However, the other two arrays, including the following array and symmetric array, can predict the simulated damage location well. The following array imaging error is within 1.2 cm even at the 150°. In addition, it is worth noting that as the focusing direction increases, it will be difficult to arrange the symmetrical arrays since the array gradually exceeds the structure. Therefore, the result for the symmetrical arrays is not given for Case 7.

## 4. Experimental Verification on an Epoxy Laminate Plate

**Figure 10:**
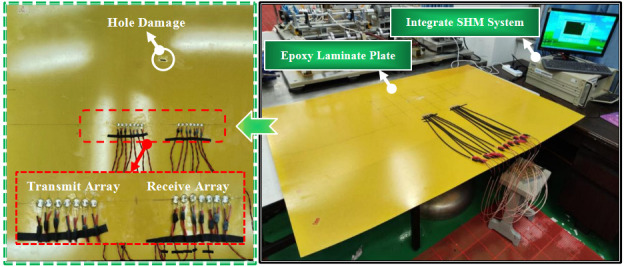
Experimental setups.

As seen in Figure 10, a thin-walled epoxy laminate plate was used in this experiment. Its size was 1000 mm × 800 mm × 2 mm. The epoxy laminate plate had a total of 16 layers, and the laying angles of each layer were 0°, 90°, and 0°, respectively. The “s” indicates that the upper and lower layers were laid symmetrically. The ply sequence was [0_2_/90_4_/0_2_]_S_ and the thickness of each ply was 0.125 mm. The integrated structural health monitoring system was applied to control the transmit and receiver of the sensor array. Eight piezoelectric sensors were pasted at equal intervals of 10 mm along the center line of the plate, which is defined as transmit array. At symmetrical positions 200 mm apart, another array of the same number and spacing was pasted as the receive array. A hole was artificially created at these polar coordinates (224 mm, 60°) as damage on the plate. The excitation frequency $f_{0}$ of the reference array element is set as 50 kHz, and the frequency increment Δf is set as 1 kHz. The sampling rate of the receive array is set to 10 MHz and the sampling time is set to 1 ms.

**Figure 11:**
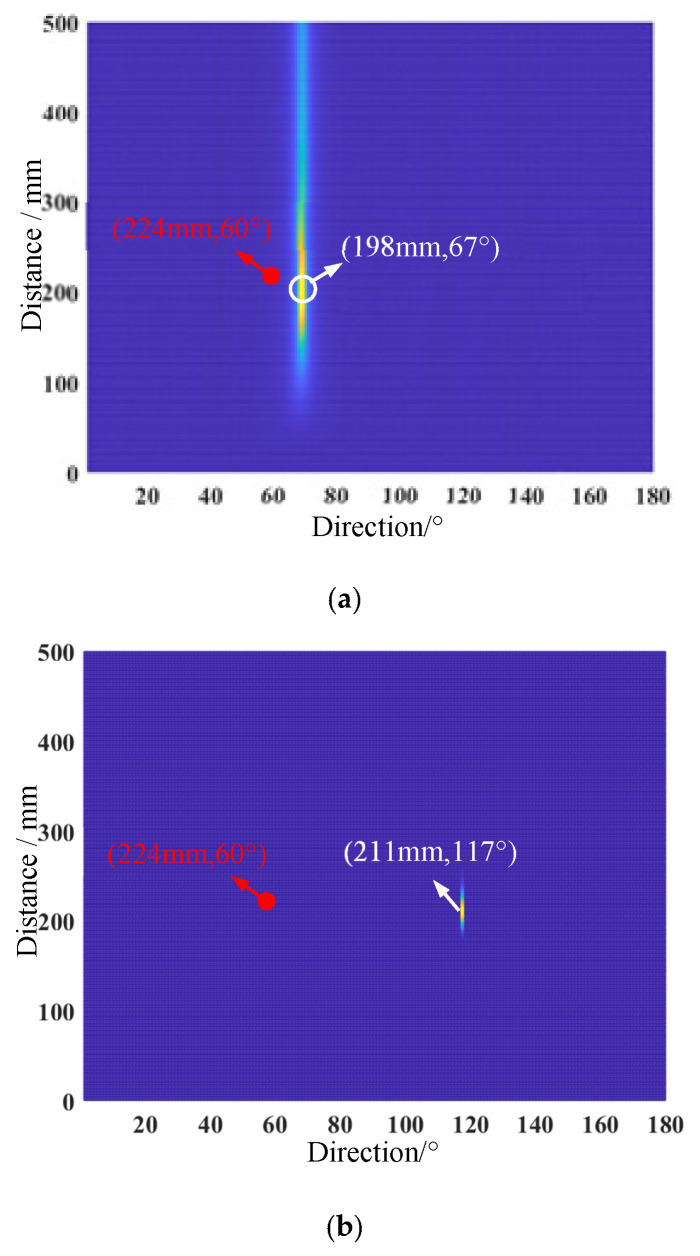
Damage imaging results of two arrays: (**a**) monostatic array; (**b**) symmetric array.

Damage imaging was performed using the two-dimensional MUSIC-based damage location method. The monostatic array is firstly used to receive the echo signals for damage imaging. As seen in Figure 11a, a bright pixel point can be easily spotted at the direction of 67°, and the peak coordinates are (198 mm, 67°). Compared with the actual damage position, there is a distance error of 2.6 cm and a direction error of 7°. The damage imaging of the symmetric array is shown in Figure 11b. The peak coordinates are (211 mm, 117°). According to Equation (18), the predicted position coordinates are (211 mm, 63°), and there is a distance error of 1.3 cm and a direction error of 3°. It can be found that the predicted damage positions of the symmetric array are better than that of the monostatic array.

## 5. Conclusions

A guided wave frequency diverse array signal model was derived with a relatively small frequency increment, which can effectively solve the beam directivity problem that is only angle-dependent in traditional ultrasonic phased arrays and can achieve distance–direction two-dimensional focusing for damage detection. Three types of receiving arrays, including a monostatic array, following array and symmetric array, were constructed to achieve the maximum damage-induced signal detection. And a two-dimensional MUSIC-based damage location method was proposed for thin-walled structures.

The validity and effectiveness of the proposed method are firstly demonstrated on an aluminum plate simulation with seven cases. The predicted damage positions of the monostatic array have a relatively large error of approximately 4 cm in the x direction and 6 cm in the y direction, and this rises sharply as the direction increases, and fails at around 150°. However, the predicted damage positions of the following array and symmetric array are all consistent with the simulated positions. The following array imaging error is within 1.2 cm, even at 150°. The validity and effectiveness of the proposed method are also experimentally demonstrated on the epoxy laminate plate. This shows that the predicted damage positions of the symmetric array are better than that of the monostatic array. The error of distance estimation is less than 1.3 cm and the error of direction estimation is less than 3°.

In further research, it would be worth performing a reliability assessment and improve the accuracy of multi-location damage detection in more complex structures.

## Figures and Tables

**Table 1:** Polar coordinates of simulated damage cases.

Damage	1	2	3	4	5	6	7
***r*** **/mm**	100	200	300	400	300	300	300
***θ/°***	105	105	105	105	120	135	150

## Data Availability

The data presented in this study are available on request from the corresponding author. The data are not publicly available due to privacy restrictions.
